# Anomalous random telegraph noise in nanoscale transistors as direct evidence of two metastable states of oxide traps

**DOI:** 10.1038/s41598-017-06467-7

**Published:** 2017-07-24

**Authors:** Shaofeng Guo, Runsheng Wang, Dongyuan Mao, Yangyuan Wang, Ru Huang

**Affiliations:** Institute of Microelectronics, Peking University, Beijing, 100871 China

## Abstract

In this paper, a new pattern of anomalous random telegraph noise (RTN), named “reversal RTN” (rRTN) induced by single oxide trap, is observed in the drain current of nanoscale metal-oxide-semiconductor field-effect transistors (MOSFETs) with high-k gate dielectrics. Under each gate voltage, the rRTN data exhibit two zones with identical amplitudes but reversal time constants. This abnormal switching behavior can be explained by the theory of complete 4-state trap model (with two stable states and two metastable states), rather than the simple 2-state or improved 3-state trap model. The results provide a direct experimental evidence of the existence of two metastable states in a single oxide trap, contributing to the comprehensive understanding of trap-related reliability and variability issues in nanoscale transistors.

## Introduction

**Figure 1:**
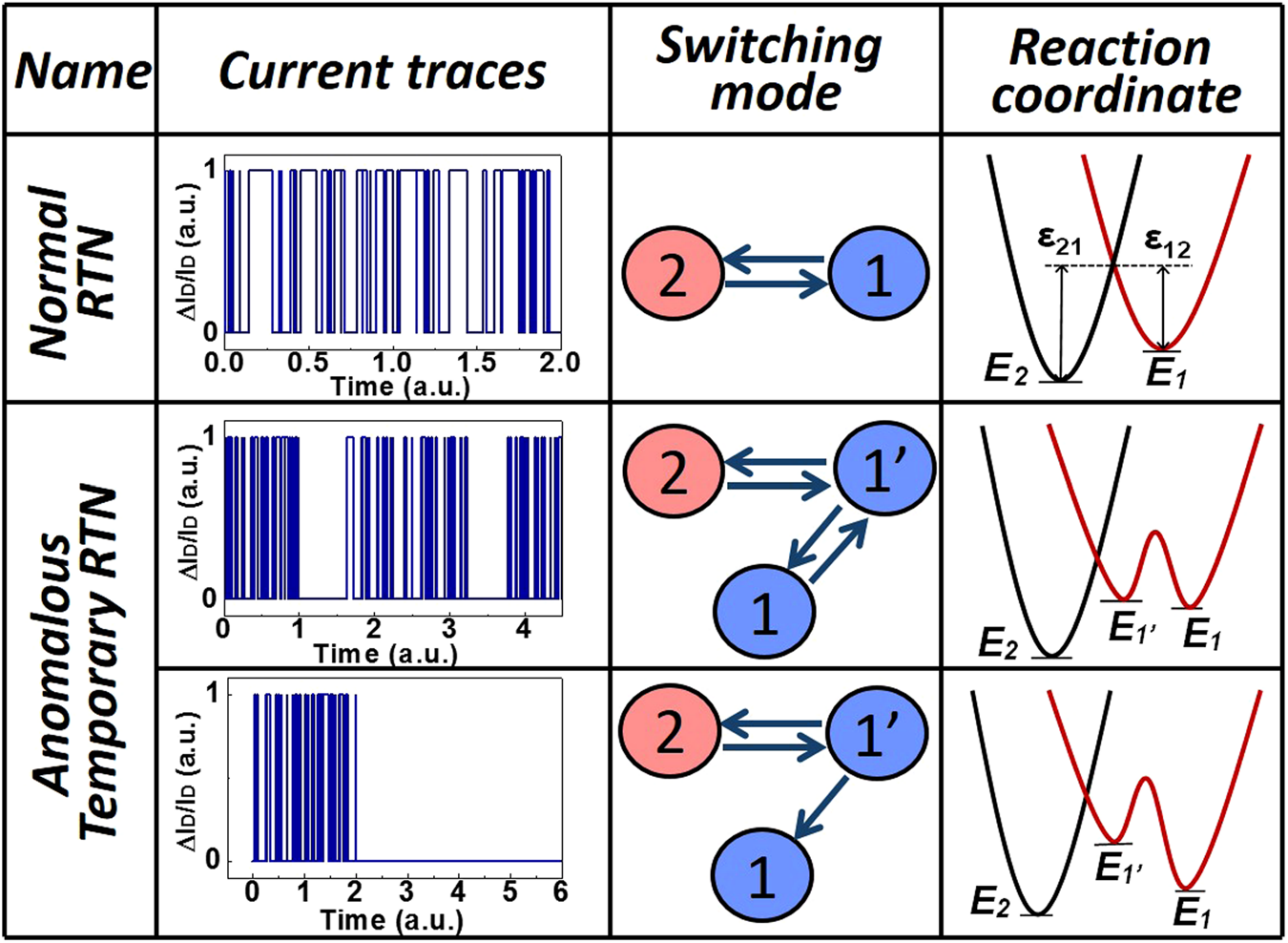
The illustrations of the current traces, switching modes and the corresponding reaction coordinate of the normal RTN and two patterns of anomalous temporary RTN^9, 10^ (tRTN), where state 2 is charged state, and state 1 and metastable state 1′ are neutral state.

With the metal-oxide-semiconductor field-effect transistor (MOSFET) dimension downscaling, oxide traps in the gate dielectric have drawn much attention due to their serious impacts on the device reliability and variability^1–11^. The random charging/discharging processes of traps have been studied directly based on the random telegraph noise (RTN) in the drain current of MOSFETs^1, 7^. Normally, the single-trap induced RTN is a typical Poisson process^7^, as illustrated in Fig. 1. The stochastic trapping/detrapping behavior can be characterized by two parameters: capture time constant (${\bar{\tau }}_{c}$) and emission time constant (${\bar{\tau }}_{e}$), representing the average waiting time to capture (or emit) a carrier from (or to) the channel^7^.

Apart from the normal RTN, two patterns of the anomalous temporary RTN (tRTN) induced by single oxide trap with two current levels have been reported^12, 13^, as also illustrated in Fig. 1, where the fast switching process can suddenly appear or disappear. Both patterns can be explained by the 3-state trap model^12, 13^, improved from the simple 2-state trap model by including one additional metastable trap state. The “quiet” phases with no switching behaviors correspond to the transition between the neutral state (1) and the neutral metastable state (1′). In order to describe the trap-induced reliability physics of bias temperature instability (BTI) in MOSFETs, recently, the complete 4-state trap model^7^ with two metastable states has also been proposed and verified with indirect proofs^7, 14, 15^, but was lack of direct evidence from RTN. In this paper, a new pattern of anomalous RTN in nanoscale MOSFETs is observed and analyzed in detail, providing a direct support on the complete 4-state trap model.

## Experimental Results

**Figure 2:**
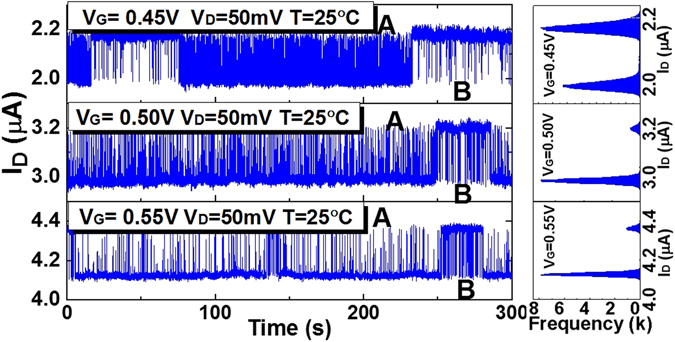
Left: Experimental results of the rRTN in the drain current of the nanoscale MOSFET under three different gate voltages. The rRTN trace exhibits two zones with different time constants. The average transition time between zone A and zone B is hundreds of seconds. Right: The histograms of the RTN data at different V_G_, indicating the two current levels.

**Figure 3:**
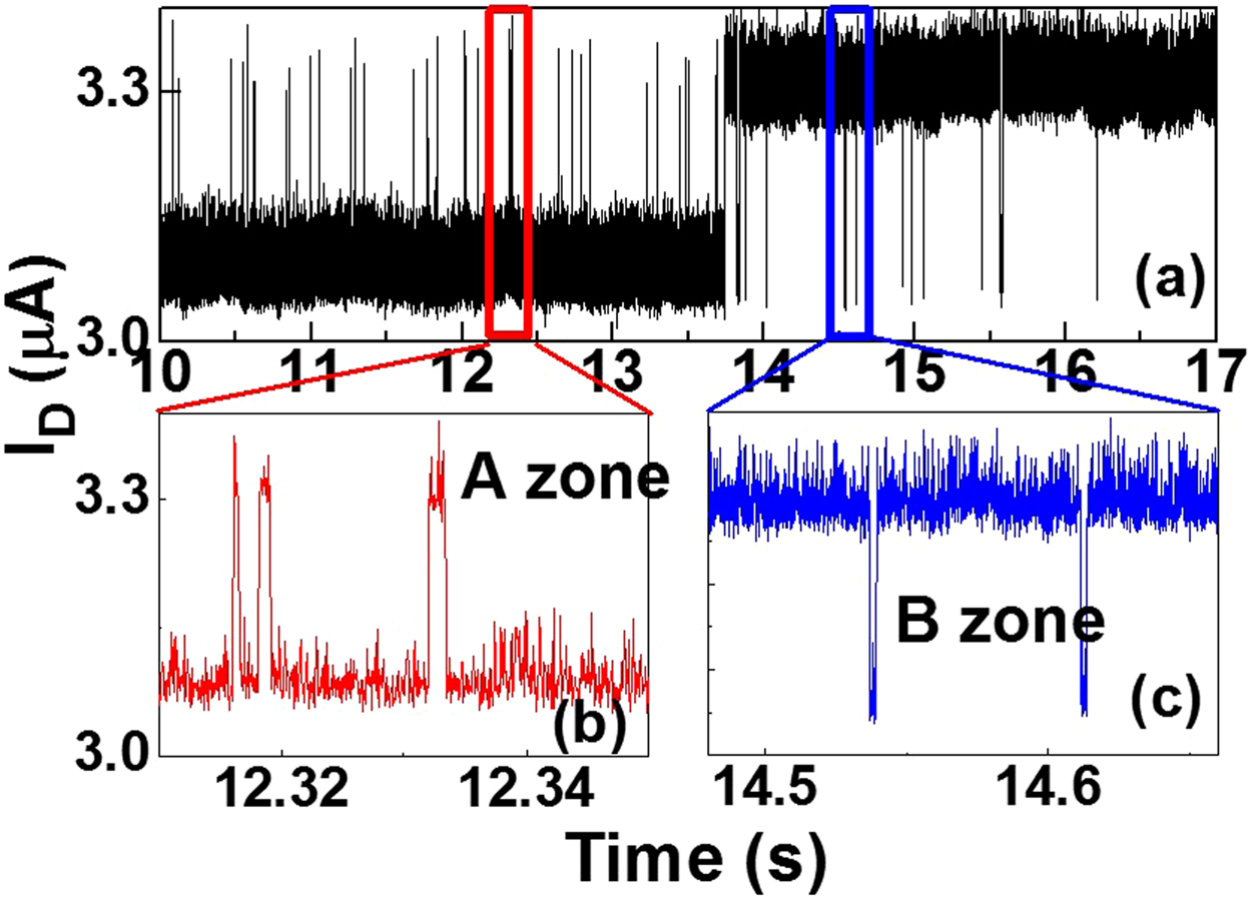
A closer view of the rRTN under *V*_*G*_ = 0.5 V in zone A and zone B separately. The emission time constant (${\bar{\tau }}_{e}$) in zone A is larger than the capture time constant (${\bar{\tau }}_{c}$), while ${\bar{\tau }}_{c}$ is larger in zone B.

Devices characterized in this work are planar high-k/metal-gate n-type MOSFETs with length L = 60 nm, width W = 300 nm and ultra-thin HfO_2_ gate dielectrics. The experimental results of the anomalous RTN in drain current under different gate voltages *V*_*G*_ are shown in Fig. 2, with the drain voltage kept at *V*_*D*_ = 50 mV, and temperature *T* = 25 °C. For other V_G_, the anomalous RTN data are shown in Fig. S1 of the supplementary materials. It can be clearly observed that this RTN exhibits two zones (zone A & zone B) under the same bias. The results are repeatable after measurement interruptions. As illustrated in Fig. 3, the expanded detail shows that the capture time constant is smaller than the emission time constant in zone A, while it has the reversal feature in zone B. Therefore, it is named “reversal RTN” (rRTN) here.

**Figure 4:**
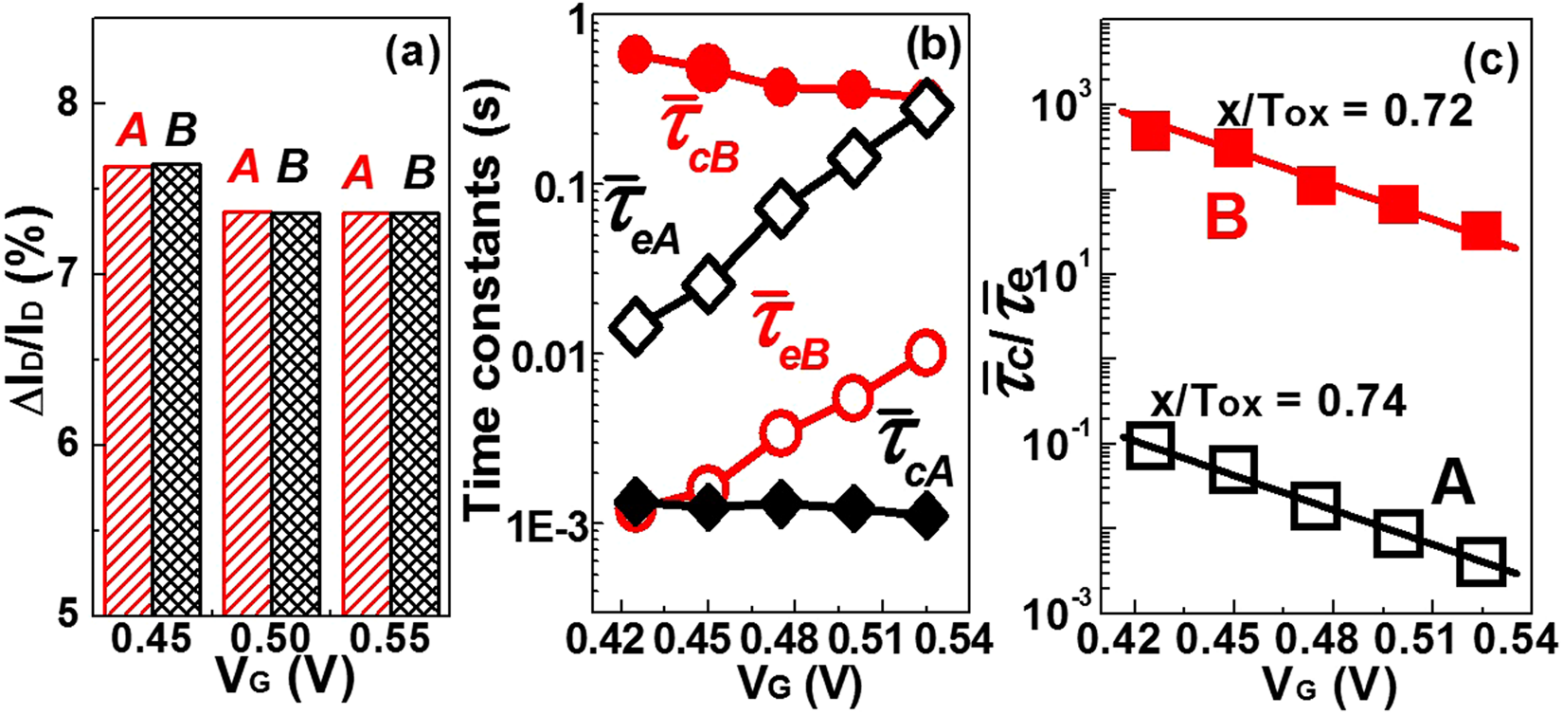
The comparisons of (**a**) amplitudes, (**b**) time constants and (**c**) the extracted trap position in zone A and zone B. The results suggest that zone A and zone B represent the same single oxide trap in the gate dielectric.

To have quantitative information, the characteristics of the rRTN in zone A and zone B are carefully investigated in the following three aspects: amplitudes, time constants and trap position. The extracted amplitudes of RTN in zone A and B are shown in Fig. 4(a), which are identical under different *V*_*G*_, indicating that the rRTN has only two current levels throughout the entire time domain. In principle, the RTN induced by *N* traps in a single device should be corresponding to 2^N^ current levels. Therefore, it is most likely that zone A and zone B represent the same trap, otherwise, there should be more than two current levels due to the superposition of RTN levels induced by more than one traps. The time constants ${\bar{\tau }}_{c}$ and ${\bar{\tau }}_{e}$ in each zone are extracted as shown in Fig. 4(b), and the extraction details are shown in Fig. S2 of the supplementary materials. Both ${\bar{\tau }}_{c}$ and ${\bar{\tau }}_{e}$ in two zones have the similar *V*_*G*_ dependence, respectively. Therefore, based on the relationship^16, 17^

1 $$\frac{x}{{T}_{OX}}=-\frac{{k}_{B}T}{q}\frac{\partial \,\mathrm{ln}\,({\bar{\tau }}_{c}/{\bar{\tau }}_{e})}{\partial {V}_{G}}$$

2 $${T}_{OX}={T}_{IL}+\frac{{\varepsilon }_{Si{O}_{2}}}{{\varepsilon }_{HK}}{T}_{HK}$$

where *T*_*IL*_ is the thickness of the SiO_2_ interfacial layer and *T*_*HK*_ the thickness of the HfO_2_ high-k layer, *ε*_*SiO2*_ and *ε*_*HK*_ are the dielectric constants for SiO_2_ and the HfO_2_ high-k dielectric, respectively, *k*_*B*_ is the Boltzmann constant and *T* is the temperature, the vertical distance *x* to the oxide/channel interface can be attained. It is worth mentioning that since the RTN measurement region is above the threshold voltage in this work, the effect of ∂ψ_S_/∂V_GS_ is small and thus is ignored for simplicity^18^. As shown in Fig. 4(c), the *x*/*T*_*OX*_ extracted from two zones are 0.74 and 0.72, respectively, which are almost the same, suggesting the same trap location in the HfO_2_ dielectric, thus providing another proof that two zones correspond to one single trap.

## Discussions on Physical Mechanism

**Figure 5:**
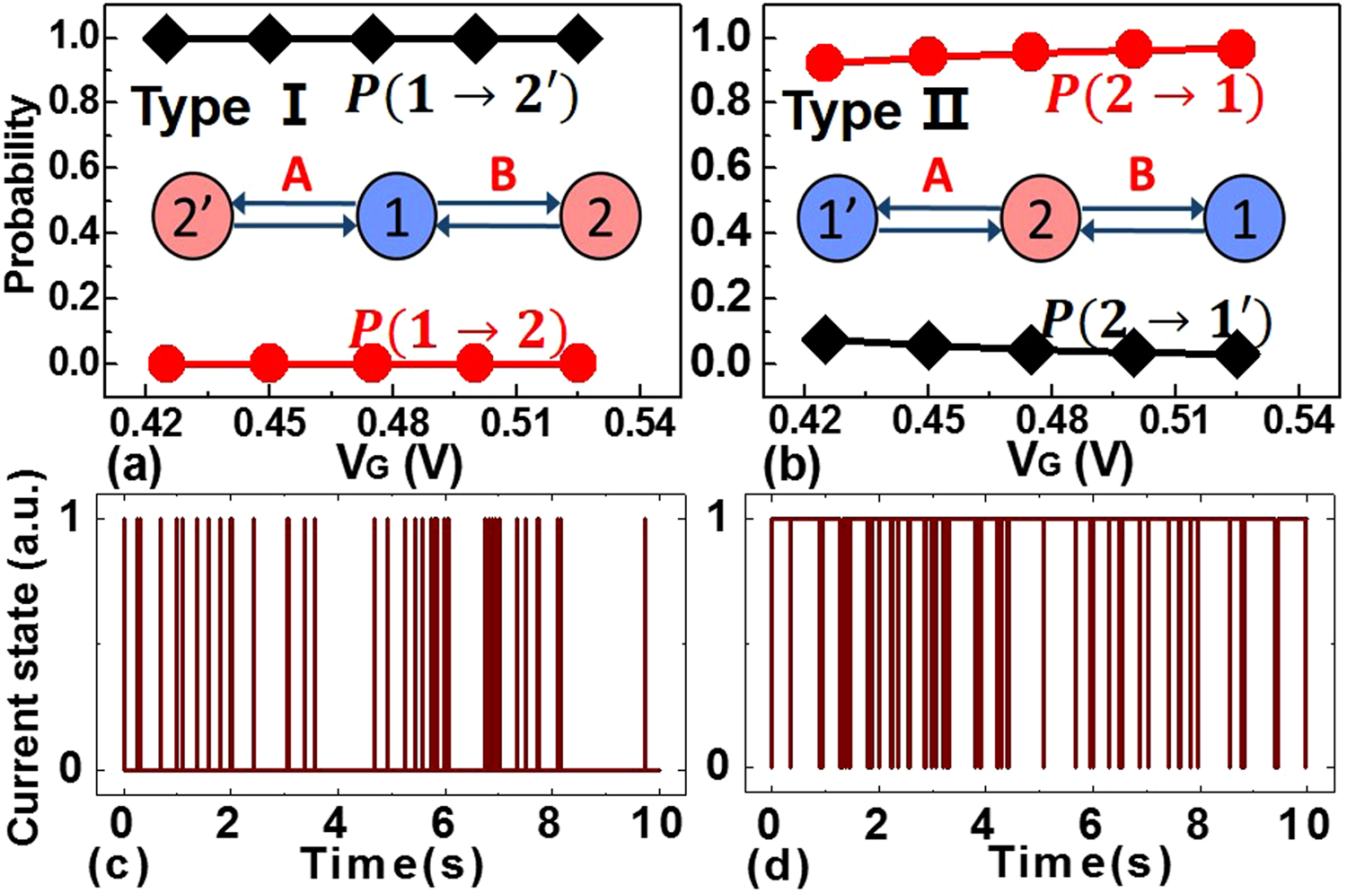
(**a**,**b**) Illustration of the transition probabilities between different states in two types (type I and type II) of the 3-state trap model, where the state 2 and metastable state 2′ are the charged states and the state 1 and metastable state 1′ are neutral states. (**c**,**d**) Theoretical simulated RTN results based on the extracted time constants with two types of 3-state trap model under *V*_*G*_ = 0.425 V.

It is believed that, during the transition from one valence state to another, the trap may go through a metastable state^1, 7, 14^. In order to explain the mechanism of rRTN with two zones, all the possible trap models, i.e., 2-state, 3-state (with one metastable state) and 4-state (with two metastable states) trap models, are studied. First, the simple 2-state trap model originating from the normal RTN theory can be excluded apparently, because metastable states should be introduced to interpret the abnormal switching phenomenon. Next, the 3-state trap model with one additional metastable state is considered. It is worth noting that the 3-state trap model as shown in Fig. 1 cannot explain the rRTN, due to the fact that the switching phenomenon in rRTN is frequent throughout the entire time domain with no “quiet” phase. Therefore, the 3-state trap model needs to be modified. There are two types of 3-state trap model can be the candidate transition modes, as shown in the inserted of Fig. 5(a,b). Based on the extracted time constants from these two zones, the transition probabilities between each two states can be calculated as follows:

For the type I:

3 $$P(1\to 2)=\frac{{\bar{\tau }}_{12^{\prime} }}{{\bar{\tau }}_{12}+{\bar{\tau }}_{12^{\prime} }}$$

4 $$P(1\to 2^{\prime} )=\frac{{\bar{\tau }}_{12}}{{\bar{\tau }}_{12}+{\bar{\tau }}_{12^{\prime} }}$$

For the type II:

5 $$P(2\to 1)=\frac{{\bar{\tau }}_{21^{\prime} }}{{\bar{\tau }}_{21}+{\bar{\tau }}_{21^{\prime} }}$$

6 $$P(2\to 1^{\prime} )=\frac{{\bar{\tau }}_{21}}{{\bar{\tau }}_{21}+{\bar{\tau }}_{21^{\prime} }}$$

The extracted transition probabilities under different *V*_*G*_ are shown in Fig. 5(a,b). For the type I model, it can be found that P(1 → 2′) is about 1, and P(1 → 2) is close to 0. It can be deduced that if the transition from state 2 to 1 is very fast, the probabilities will lead to almost the same results as normal RTN with two current levels. If the transition from 2 to 1 is slow, there will be a long time period when the current is staying at the low level. Based on the extracted time constants, the RTN simulation with multiple states can be performed. The details of the theoretical simulation method are shown in Figs S3 and S4 in the supplementary materials, which is similar to ref. 19. The result is shown in Fig. 5(c). As expected, the 3-states model of type I are inconsistent with the experimental results. Similarly, the type II model also fails to interpret the experimental rRTN results, which is verified by the simulated RTN result shown in Fig. 5(d).

**Figure 6:**
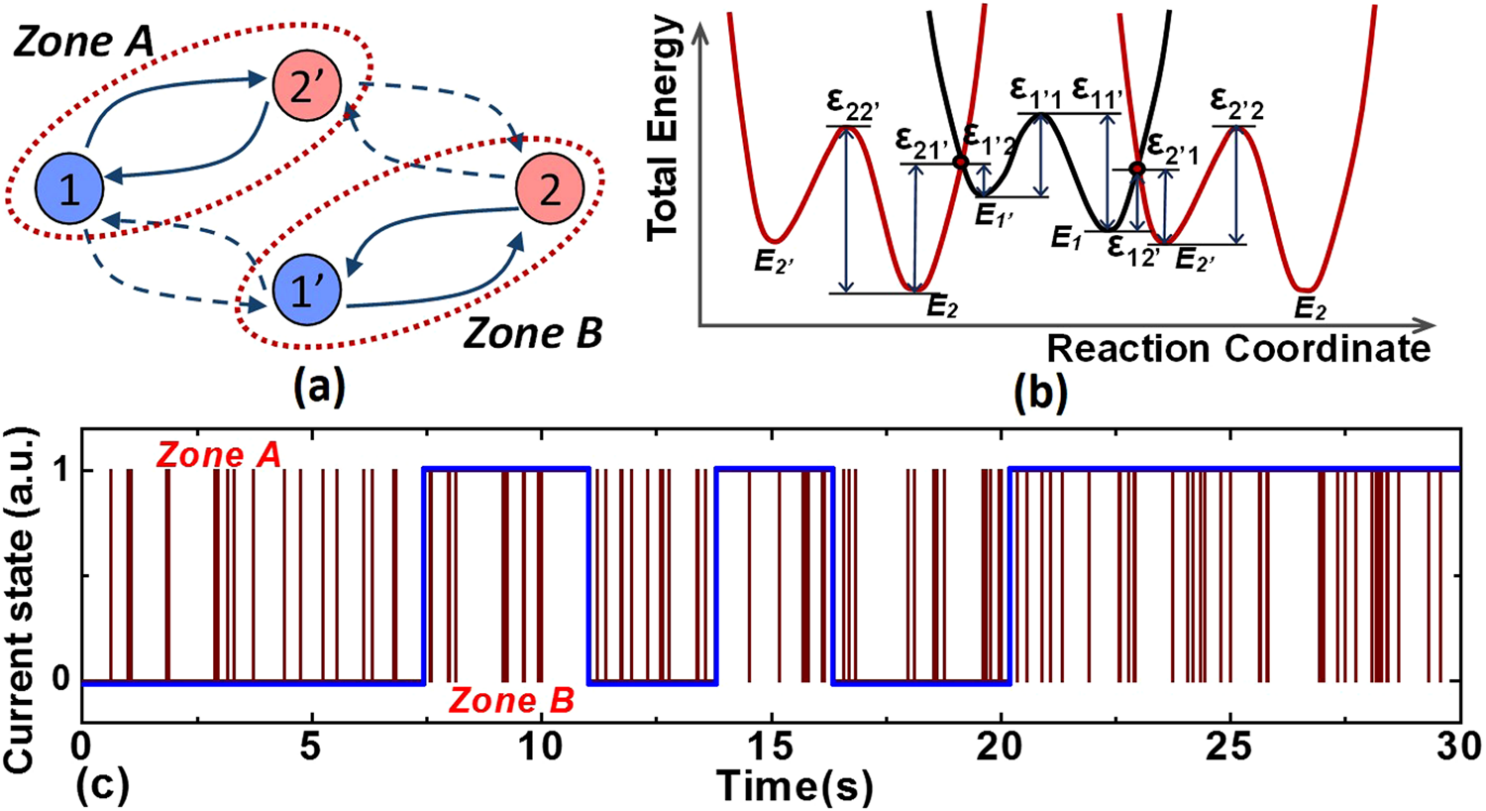
(**a**) Illustration of the complete 4-state model. The zone A and zone B in rRTN correspond to the transition processes in circles. (**b**) Schematic of the corresponding reaction coordinate of the complete 4-state trap model. (**c**) Theoretical simulated RTN results based on the extracted time constants associated with the complete 4-state trap model, which are consistent with the experimental results in Fig. 2.

Last, the complete 4-state trap model with two metastable trap states is examined, as shown in Fig. 6(a), including one neutral metastable state (1′) and one charged metastable states (2′). Zone A corresponds to the switching process between state 1′ and state 2, and zone B is between state 1 and state 2′. It is worth mentioning that the transition probabilities of 1 → 1′ (or 2 → 2′) is usually smaller compared with other transition processes, due to higher barrier ε_11′_ (or ε_2′2_) than others. Therefore, in previously reported experiments, it is rare to observe RTN data featuring the transitions among four states. Regarding the rRTN observed in this paper, particularly, the 4-state trap model can be described in the reaction coordinate as shown in Fig. 6(b). The transition barriers should follow these relationships: ε_12′_ < ε_11′_ and ε_2′1_ < ε_2′2_, otherwise, there is only single zone, as illustrated based on the simulated RTN results in Fig. S5 of the supplementary materials. Therefore, the switching between 1 ↔ 2′ (or, 2 ↔ 1′) are preferred rather than between 1 ↔ 1′ (or, 2 ↔ 2′). Once the trap state turns to the zone A, it will stay in this zone for a period until the state turns to 1′ via 1 (or, to 2 via 2′). Similarly, it can also stay in the zone B for a period until the trap state turn to 1 via 1′ (or, to 2′ via 2). In other words, the fast switching zones A and B can appear randomly with a lasting time longer than the average transition time, which is exactly consistent with the observed rRTN. Based on the extracted time constants, the simulated RTN result with the 4-state trap model can be obtained, as shown in Fig. 6(c), which is consistent with the rRTN experimental results. Finally, it can be concluded that the experimental results can only be explained by the 4-state trap model (with two metastable states), rather than the simple 2-state trap model (without metastable states) and the improved 3-state model (with one metastable state). In addition, as mentioned earlier, if the trap exhibits four states rather than two states, it can affect the frequency dependence of BTI reliability^15, 20, 21^, which is still under investigation.

## Conclusion

In summary, single-trap induced rRTN with two evident zones is observed in nanoscale transistors with HfO_2_ gate oxide. Based on the analysis of transition probabilities and the corresponding simulated RTN results, the abnormal transition mechanism can be explained by the complete 4-state trap model, thus directly verifying the existing of two metastable states at single-trap based on the experimental results, which is helpful for comprehensive understanding of trap-related device reliability and variability physics.

## Electronic supplementary material

Supplementary Information
