# Supplementary material for: Anomalous random telegraph noise in nanoscale transistors as direct evidence of two metastable states of oxide traps
Source: Sci Rep. 2017 Jul 24;7:6239. doi: 10.1038/s41598-017-06467-7 (PMC5524939; doi:10.1038/s41598-017-06467-7)
Supplement: Supplementary file 1 — Supplementary Information [file 41598_2017_6467_MOESM1_ESM.pdf]

Anomalous random telegraph noise in nanoscale transistors as direct evidence of two metastable states of oxide traps

Supplementary Materials

Shaofeng Guo, Runsheng Wang*, Dongyuan Mao, Yangyuan Wang and Ru Huang

Institute of Microelectronics, Peking University, Beijing, 100871, China

*E-mail: r.wang@pku.edu.cn

Supplementary Fig. S1 Experimental results of the rRTN in the drain current under other gate voltages.

Supplementary Fig. S2 Illustrations of the extracted (a) capture and (b) emission times.

Supplementary Fig. S3 Simulation flow of the normal RTN with two stable states.

Supplementary Fig. S4 Simulation flow of the RTN accompanied with 3-state trap model.

Supplementary Fig. S5 Simulated RTN results under all the possible cases.

Fig. S1

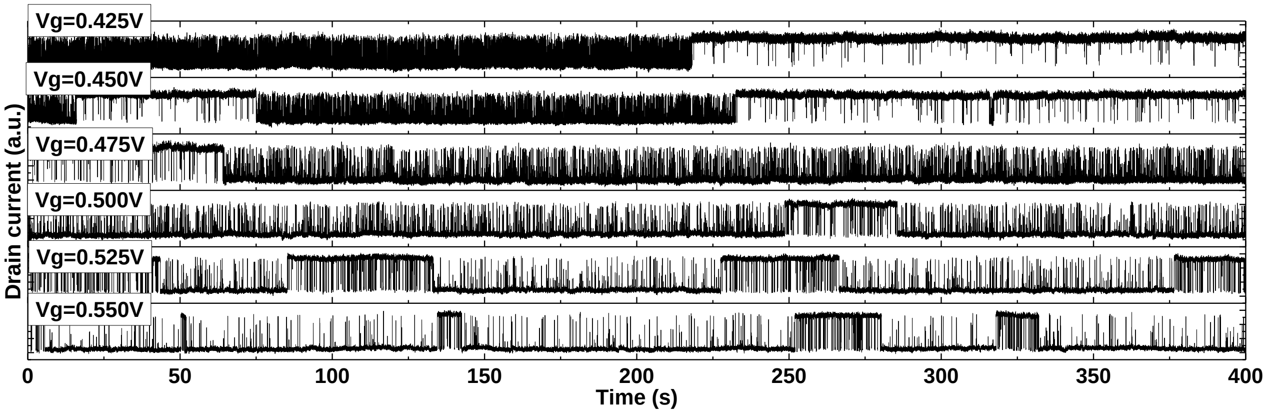

Supplementary Fig. S1 Experimental results of the rRTN in the drain current under other gate voltages. It is worth noting that, for much larger or much smaller V_G, it is beyond the RTN test window of the measurement system due to its limited sampling rate and/or limited memory depth.

Fig. S2

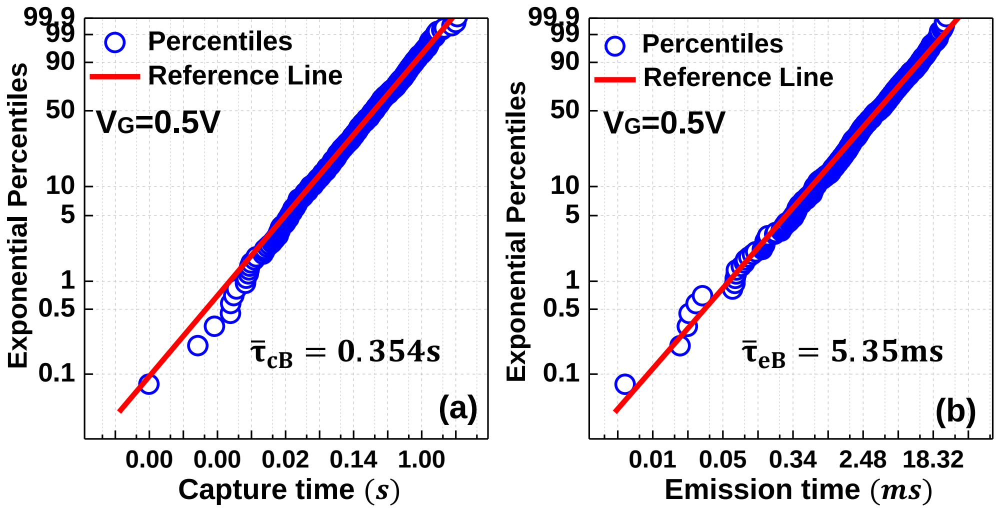

Supplementary Fig. S2 Illustrations of the extracted (a) capture and (b) emission times.

In this rRTN data, each time to capture/emit ($\tau_{c,i}/\tau_{e,i}$) a carrier from/to the channel can be recorded according to the step-like switching signals, due to the clear separation of the high and low current levels. The time constants of RTN can be extracted by fitting the exponential distributions of the capture and emission times, as shown in Fig. S2(a)&(b) for example. In addition, since it is exponential distribution, the time constants can also be extracted by directly averaging the statistical capture and emission times. For example, the results are $\bar{\tau}_{cB} = 0.360s$ and $\bar{\tau}_{eB} = 5.49ms$ for the data in the figures, which are almost identical to the results from the fitting method.

Fig. S3&S4

Basically, the random trapping/detrapping processes of normal 2-state RTN with two states follow the Markov process [1,7], which can be simulated based on the following flow (Fig. S2). where *rand* is a random number with the uniform distribution between 0 and1, Pc=$\Delta t/\bar{\tau}_c$ and Pe=$\Delta t/\bar{\tau}_e$. More detailed information about the MC simulation can be found in [19].

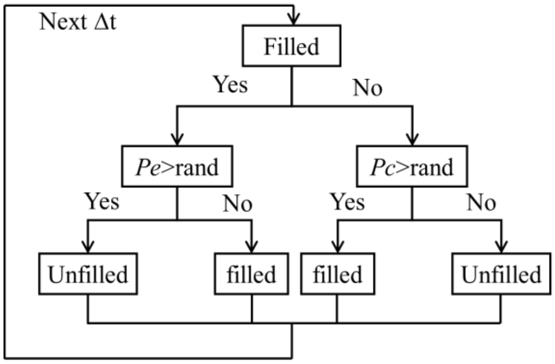

Supplementary Fig. S3 Simulation flow of the normal RTN with two stable states.

For the RTN with 3-state or 4-state trap model, similar simulation methods are used. Here, we take the RTN with 3-state trap model, i.e., 2'(filled)↔1(unfilled)↔2(filled), for an example, as shown in the following flowchart (Fig. S3), where *Pc*(2)=$\Delta t/\bar{\tau}_{c12}$, *Pc*(2')=$\Delta t/\bar{\tau}_{c12'}$, *Pe*(2'→1)=$\Delta t/\bar{\tau}_{e2'1}$, *Pe*(2→1)=$\Delta t/\bar{\tau}_{e21}$, *rand* and *rand'* are random numbers in the uniform distribution between 0 and 1.

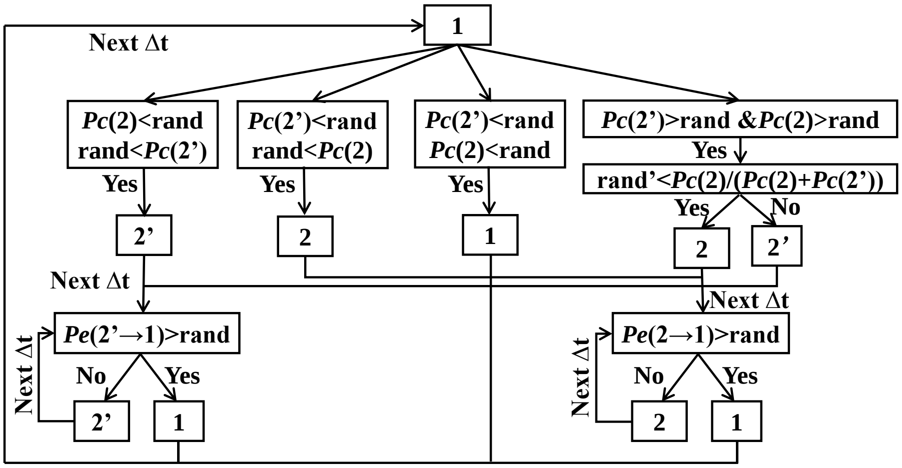

Supplementary Fig. S4 Simulation flow of the RTN accompanied with 3-state trap model.

For the RTN simulation with 4-state trap model, the simulation method is similar to the above flow.

Fig. S5

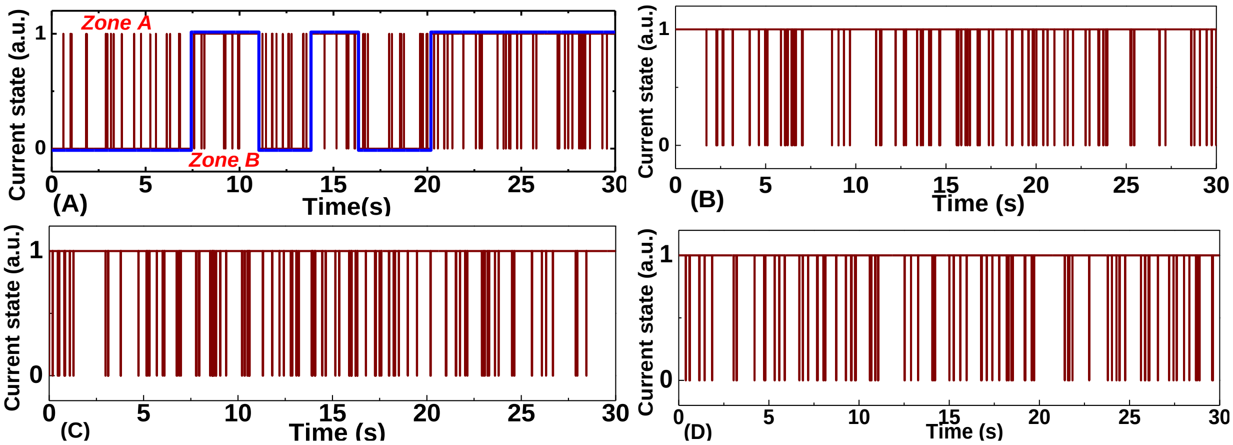

Supplementary Fig. S5 Simulated RTN results under all the possible cases.

Considering the relationship between $\varepsilon12'$ and $\varepsilon11'$, and the relationship between $\varepsilon2'1$ and $\varepsilon2'2$, there can only be four possible cases: (A): $\varepsilon12'<\varepsilon11'$& $\varepsilon2'1<\varepsilon2'2$; (B): $\varepsilon12' >\varepsilon11'$& $\varepsilon2'1<\varepsilon2'2$; (C): $\varepsilon12' <\varepsilon11'$& $\varepsilon2'1>\varepsilon2'2$; (D): $\varepsilon12' <\varepsilon11'$& $\varepsilon2'1<\varepsilon2'2$. For each case, we performed RTN simulations, as shown in Fig. S4, indicating only case A is able to reproduce the two-zone phenomenon as observed in the experiments. Therefore, based on the above analysis, we can draw the conclusion ($\varepsilon12'<\varepsilon11'$ and $\varepsilon2'1<\varepsilon2'2$) for the rRTN in this work.
